# Molecular, clinicopathological, and immune correlates of *LAG3* promoter DNA methylation in melanoma

**DOI:** 10.1016/j.ebiom.2020.102962

**Published:** 2020-08-30

**Authors:** Anne Fröhlich, Judith Sirokay, Simon Fietz, Timo J. Vogt, Jörn Dietrich, Romina Zarbl, Mike Florin, Pia Kuster, Gonzalo Saavedra, Susana Ramírez Valladolid, Friederike Hoffmann, Lukas Flatz, Sandra S. Ring, Carsten Golletz, Torsten Pietsch, Sebastian Strieth, Peter Brossart, Gerrit H. Gielen, Glen Kristiansen, Friedrich Bootz, Jennifer Landsberg, Dimo Dietrich

**Affiliations:** a Department of Dermatology and Allergy, University of Bonn, Bonn, Germany; b Department of Otolaryngology, Head and Neck Surgery, University Hospital Bonn, Sigmund-Freud-Str. 25, 53105 Bonn, Germany; c Institute of Pathology, University Hospital Bonn, Bonn, Germany; d Institute of Neuropathology, University Hospital Bonn, Bonn, Germany; e Institute of Immunobiology, Kantonsspital St Gallen, St Gallen, Switzerland.; f Department of Oncology and Haematology, Kantonsspital St Gallen, St Gallen, Switzerland; g Department of Dermatology, University Hospital Zurich, Zurich, Switzerland; h Department of Dermatology and Allergology, Kantonsspital St Gallen, St Gallen, Switzerland; i Microbiology and Immunology PhD Program, University of Zurich, Zurich, Switzerland; j Department of Oncology, Hematology and Rheumatology, University Hospital Bonn, Bonn, Germany

**Keywords:** *LAG3*, DNA Methylation, Melanoma, Predictive Biomarker, Immunotherapy

## Abstract

**Background:**

The co-receptor lymphocyte-activation gene-3 (LAG3, LAG-3, CD223) is a potential target for immune checkpoint inhibition immunotherapies. However, little is known about the biological and clinical significance of *LAG3* DNA methylation in melanoma and its microenvironment.

**Methods:**

We evaluated *LAG3* promoter and gene body methylation in a cohort of *N* = 470 melanoma patients obtained from The Cancer Genome Atlas (TCGA cohort), an independent cohort of *N* = 120 patients from the University Hospital Bonn, and in subsets of peripheral blood leukocytes, melanocytes, and melanoma cell lines. We validated the association of *LAG3* methylation with mRNA expression *in vitro* in the melanoma cell line A375 treated with the hypomethylating agent 5-azacytidine and stimulated with interferon-γ. Finally, we investigated correlations between *LAG3* methylation and progression-free survival in patients treated with immune checkpoint blockade (ICB cohort, *N* = 118).

**Findings:**

Depending on the analysed locus (promoter, gene body) we found region-dependent significant *LAG3* methylation differences between monocytes, B cells, CD8^+^ and CD4^+^ T cells, regulatory T cells, melanocytes, and melanoma cell lines. In tumor tissues, methylation correlated significantly with *LAG3* mRNA expression, immune cell infiltrates (histopathologic lymphocyte score and RNA-Seq signatures of distinct immune infiltrates), and an interferon-γ signature. Finally, *LAG3* methylation was associated with overall survival in the TCGA cohort and progression-free survival in the ICB cohort. We detected basal *LAG3* mRNA expression in the melanoma cell A375 and an interferon-γ inducible expression after demethylation with 5-azacytidine.

**Interpretation:**

Our study points towards an epigenetic regulation of *LAG3* via promoter methylation and suggests a prognostic and predictive significance of *LAG3* methylation in melanoma. Our results give insight in the tumor cell-intrinsic transcriptional regulation of *LAG3* in melanoma. In perspective, our results might pave the way for investigating *LAG3* methylation as a predictive biomarker for response to anti-LAG3 immune checkpoint blockage.

**Funding:**

A full list of funding bodies that contributed to this study can be found in the Acknowledgements section.

**Research in context**

**Evidence before this study**

The inhibitory receptor LAG3 (lymphocyte-activation gene 3; CD223, LAG-3) is an attractive new target for cancer immunotherapy and currently LAG3-targeted antibodies are tested in clinical trials in diverse malignancies including melanoma. So far, knowledge about epigenetic regulation of *LAG3* and tumor cell-intrinsic expression of *LAG3* in melanoma is scarce. However, gaining deeper insight in regulatory mechanisms of immune checkpoints, especially on the epigenetic level, is an important prerequisite for development of precise predictive biomarkers and therapeutic strategies.

**Added value of this study**

Our study presents an in-depth analysis of *LAG3* methylation in melanoma based on data of a recent landscape paper of The Cancer Genome Atlas Network and two additional melanoma cohorts, including one cohort of patients treated with immune checkpoint inhibitors. Functional analyses in melanoma cell lines and correlation of *LAG3* methylation data with clinicopathological and immunological features substantiate our findings*.* Our study demonstrates a tumor cell-intrinsic mRNA expression of *LAG3*, which is regulated via DNA methylation. Our results provide valuable insights in the prognostic significance of *LAG3* in melanoma. Additionally, we present first evidence for *LAG3* DNA methylation as a predictive biomarker for response to immune checkpoint inhibitors in melanoma.

**Implications of all the available evidence**

Our data demonstrate the significance of tumor cell-intrinsic *LAG3* expression in melanoma and provide a rationale for investigating *LAG3* methylation as a prognostic and predictive biomarker in melanoma. Our findings point to *LAG3* DNA methylation as a predictive biomarker in patients receiving immune checkpoint blocking agents and may thus assist personalized therapeutic decision making.

Alt-text: Unlabelled box

## 1 Introduction

With the advent of immune checkpoint blockade (ICB) immunotherapy of cancer has become a major pillar in the treatment of advanced cancers, among them melanoma, lung cancer, renal cell carcinoma, and hematologic malignancies [1]. Most of the insights into the treatment with checkpoint inhibitors have been gained from malignant melanoma where the blockade of the PD-1 and CTLA-4 are in clinical routine for the treatment of metastasized melanoma for more than five years and have meanwhile also been approved in the adjuvant setting (adjuvant CTLA-4 is approved by the FDA only). A major clinical challenge in the treatment of advanced melanoma with ICB is the development of resistant relapsing disease or primary resistance to therapy. To overcome or even prevent therapy resistance additional immune checkpoint inhibitory receptors are evaluated as targets of immunotherapy. The inhibitory receptor LAG3 (lymphocyte-activation gene 3, CD223) is a promising candidate and is currently considered as a potential new target.

At present, several clinical phase II and III studies investigate LAG3 targeting agents (e.g. relatlimab, Bristol Myers Squibb, New York City, NY, USA), as well as ideal therapeutic sequences and combinations of LAG3 antibodies with agents targeting PD-1 and CTLA-4 in several malignancies including melanoma. Beyond, a dual checkpoint inhibitor targeting CTLA-4 and LAG3 and bispecific antibodies targeting PD-1 and LAG3 are tested in clinical trials. Several more LAG3 targeted therapies are in preclinical development, aimed against cancer but also against autoimmune diseases.

LAG3 is a type I transmembrane receptor that is mostly expressed on activated T cells and natural killer (NK) cells. It has been shown to predominantly interact with MHC class II molecules. Other described ligands are galectin 3, LSECtin [2], and fibrinogen-like protein 1 (FGL-1) [3]. Beyond the expression on T cells and NK cells, LAG3 is constitutively expressed on plasmacytoid dendritic cells (DCs) [4], whereas no expression is described for myeloid or lymphoid DC subsets [5]. The influence of LAG3 on NK cells, T cells, and plasmacytoid DCs is so far not completely understood [6]. Regulatory T cells (Tregs) express LAG3 in dynamic levels, depending on the state of activation. High levels of LAG3 have been found on immunosuppressive Tregs in cancer patients [7], e.g. in melanoma and colorectal carcinoma [8]. In addition, a recent report suggests a LAG3 expression by tumor cells as shown in clear cell renal cell carcinomas [9].

The role of LAG3 in cancer immunology has been implicated in negative regulation of T cell responses and – together with PD-1 – in T cell exhaustion, facilitating tumor escape [10]. Sustained T cell activation in malignant diseases or chronic inflammation leads to consistent co-expression of LAG3 on T cells, together with other inhibitory receptors, among them PD-1, TIM-3, and TIGIT [11]. The inhibitory functions of LAG3 include impaired proliferation of T cells and cytokine production including interferon gamma (IFN-γ) and tumor necrosis factor alpha (TNFα) [7,[12], [13], [14], [15], [16], [17]]. LAG3 interaction with its ligands galectin 3 and LSECTin inhibits IFN-γ secretion *in vitro* [18] and in melanoma cells [19]. Beyond, LAG3 signaling has also been shown to modulate autoimmunity [20].

Regulation of LAG3 expression on the protein level takes place by cleavage via ADAM10 resulting in shedding of sLAG3 or by storage and degradation in lysosomes [21]. A prognostic significance for LAG3 expression has been described in various malignancies [22], [23], [24], [25], [26], [27], [28]. However, reports on the prognostic value of LAG3 expression are controversial, depending on the specific tumor entity. High expression of LAG3 on tumor infiltrating lymphocytes and the presence of its soluble form (sLAG3) in the serum of patients has been associated with a better prognosis in some types of cancer, including hormone receptor positive breast cancer [22,28], gastric cancer [27], and colon cancer [26]. On the opposite, high expression of MHC class II as a ligand for LAG3 on tumor cells seems to portend a poorer prognosis and may indicate exhaustion of tumor infiltrating T cells in melanoma [29]. A correlative analysis in clear cell renal cell carcinoma demonstrated prognostic significance for *LAG3* methylation and mRNA expression, with high LAG3 expression indicating adverse overall survival [9]. This result was confirmed in other studies on metastatic renal cell cancer [24]. Beyond, LAG3 expression was related with poor survival in non-small cell lung cancer [25] and head and neck squamous cell carcinoma [23]. A recent meta-analysis of 15 studies investigated the prognostic value of LAG3 in cancer [30]. However, no study enclosing melanoma patients was included. The authors claimed LAG3 expression to be associated with better overall survival. The trend toward an association of LAG3 expression with outcome was higher in early stage cancer than in metastatic disease. The controversial findings on the prognostic significance of LAG3 reflect the complex role of LAG3 within the orchestra of immune response.

Epigenetic regulation mechanisms, including DNA methylation, have an elementary function in T cell differentiation and exhaustion [31], [32], [33] and have been demonstrated to modulate immune checkpoint genes. Hypomethylation of the immune checkpoint *CTLA4* has already been suggested as a biomarker for T cell exhaustion [34] and for response to immunotherapy [35]. Correlations between methylation status and the expression of immune checkpoint molecules and its prognostic significance have been demonstrated for *PD-1, PD-L1*, and PD-1 ligand 2 (*PD-L2*) in various malignancies, among them melanoma [32,[36], [37], [38], [39], [40], [41], [42], [43], [44]]. Other studies confirmed the epigenetic regulation of immune checkpoint expression, including LAG3, via DNA methylation in breast and colorectal cancer [45,46]. Recently, we could identify DNA methylation of the immune checkpoint tumor necrosis factor receptor super family member 9 (*4-1BB, TNFRSF9*) as a predictive biomarker for response to immunotherapy in melanoma patients [47].

So far, little is known about the epigenetic regulation of the *LAG3* gene that is located on chromosome 12 in close proximity to the gene that encodes CD4 [48]. This study aims to investigate the association of *LAG3* DNA methylation with gene expression, clinicopathological parameters, molecular and immune correlates, and outcome in melanoma. Doing this, we tested *LAG3* methylation as a prognostic biomarker and as a predictive biomarker for response to immune checkpoint blockade in melanoma. Insights into the epigenetic regulation of this gene might pave the way to develop predictive biomarkers to identify patients potentially benefitting from a treatment with LAG3-antagonists.

## 2 Methods

### 2.1 Ethics statement

Data generation by the TCGA Research Network was performed in accordance with the Helsinki Declaration of 1975. Patient inclusion and sample analyses at the University Hospital Bonn were approved by the Institutional Review Board (IRB) of the University Hospital Bonn (vote 187/16). The study received ethical approval from the Ethikkommission Ostschweiz (Project ID 2016-009918) and was conducted accordingly. Informed consent was obtained from all individual participants included in the study.

### 2.2 Patients and cell lines

*TCGA cohort:* The results reported here are partly based on data generated by The Cancer Genome Atlas Research Network (TCGA, http://cancergenome.nih.gov/). A total of *N* = 470 samples from the TCGA skin cutaneous melanoma (SKCM) cohort were included. We included only primary solid and metastatic tumor tissue samples. One sample per patient was analyzed and primary solid tumor tissues were included from those patients who provided both, primary solid and metastatic tumor tissues. Clinicopathological data were obtained from the TCGA Research Network. Molecular data was adopted from a study previously published by the TCGA Research Network [49]. Clinicopathological data and molecular data are summarized in Supplemental Table 1. Sample purity and ploidy estimates provided by the TCGA Research Network were calculated using the ABSOLUTE algorithm [50]. The leukocyte fraction within the tumor samples was quantified by Saltz *et al.* and Thorsson *et al.* who used DNA methylation array data to quantify leukocytes [51,52]. We additionally used the results provided by Thorsson *et al.* [52] who calculated RNA-Seq signatures as estimates for distinct immune cell infiltrates using the CIBERSORT algorithm [53]. Further data on infiltrating lymphocytes were again adopted from the TCGA Research Network [49]. Data on lymphocyte distribution (0-3; 0 = no lymphocytes within the tissue, 1 = lymphocytes present involving <25% of the tissue cross sectional area, 2 = lymphocytes present in 25 to 50% of the tissue, 3 = lymphocytes present in >50% of tissue), lymphocyte density (0-3; 0 = absent, 1 = mild, 2 = moderate, 3 = severe), and lymphocyte score (0-6, score defined as the sum of the lymphocyte distribution and density scores) were adopted from TCGA Research Network [49]. Informed consent was obtained by the TCGA Research Network from all patients in accordance with the Helsinki Declaration of 1975 [49].

*Validation cohort, UHB cohort*: In the validation analysis tumor tissue samples of *N* = 120 melanoma patients of the University Hospital Bonn (UHB cohort) were included. The cohort comprised tissue obtained from primary melanomas, subcutaneous and cutaneous metastases, and lymph node metastases. The tumor tissues were obtained from treatment-naïve patients (no systemic anti-tumor treatment, including targeted therapies or immune checkpoint blockade in the therapeutic or adjuvant setting). Tumor-infiltrating lymphocytes (TILs) in the validation analysis (UHB cohort) were assessed using the Clark scoring system [54].

*Immune checkpoint blockade cohort, ICB cohort*: We included tumor tissue samples from *N* = 118 melanoma patients treated with immunotherapy in the University Hospital Bonn and the Kantonsspital St Gallen, Switzerland. The tissue had been obtained before initiation of the immunotherapy, therefore melanoma samples included were naïve to systemic therapies, including the adjuvant setting. The ICB cohort consists of 45 (38.1%) female and 73 (61.9%) male (a total of *N* = 118) late-stage melanoma patients with a median age of 70 years (range: 28 – 92). First-line immunotherapy included 68 (57.6%) anti-PD-1 monotherapy, 24 (20.3%) anti-CTLA-4 monotherapy, 25 (21.2%) combined anti-PD-1 / anti-CTLA-4 immunotherapy, and one (0.6%) combined anti-PD-1 / anti-IDO immunotherapy. According to Response Evaluation Criteria in Solid Tumors (RECIST) version 1.1, best objective response included 19 (16.1%) patients with complete response (CR), 34 (28.8%) with partial response (PR), 6 (5.1%) with stable disease (SD), and 57 (48.3%) with progressive disease (PD). The sample collection and inclusion of the patient cohort to our study was approved by the Institutional Review Board (IRB) of the University Hospital Bonn and the Institutional Review Board (IRB) Ostschweiz.

*Cell lines and isolated immune cells:* Data from 16 melanoma cell lines and 23 melanocyte cell lines were downloaded from NCBI (National Center for Biotechnology Information, Bethesda, MD, USA) Gene Expression Omnibus (Gene Expression Omnibus (GEO) accession numbers: melanoma cell lines: GSE51547 (*N* = 9), GSE95816 (*N* = 7); melanocytes: GSE51547 (*N* = 6), GSE44662 (*N* =3), GSE86355 (*N* = 14)) [55], [56], [57]. Results from isolated immune cells (*N* = 97 CD4^+^ T cells, *N* = 24 CD8^+^ T cells, *N* = 18 Tregs, *N* = 60 B cells, and *N* = 53 monocytes) were obtained from three previous studies which included 26 healthy controls from Scotland (GSE87650), six healthy Israeli women (GSE71245), and 72 healthy American volunteers (GSE59250) [58], [59], [60]. The human melanoma cell line A375 for cell culture experiments as described below was purchased from American Type Culture Collection (ATCC, Manassas, VA, USA)

### 2.3 Cell culture

We used the human melanoma cell line A375 for investigating *LAG3* mRNA expression in melanoma cells. A375 cells were grown adherent and maintained in complete RPMI 1640 medium (Gibco, Life Technologies, UK) supplemented with 10% fetal calf serum (Pan Biotech, Aidenbach, Germany), 2 mM L-glutamine (Life Technologies, Carlsbad, CA, USA), 10 mM non-essential amino acids (Life Technologies), 1 mM HEPES (4-(2-hydroxyethyl)-1-piperazineethanesulfonic acid; Life Technologies), 20 μM 2-mercaptoethanol (Sigma-Aldrich, St. Lois, Missouri, USA), 100 U/ml penicillin, and 100 μg/ml streptomycin (Gibco).

Melanoma cells were either left untreated for 72 h or treated with IFN-γ or demethylating 5-azacytidine (5‐aza‐2‐deoxycytidine, 5‐aza‐dC; abcam, Cambridge, UK), or both, 5‐aza‐dC and IFN-γ. For 5‐aza‐dC treatment, 10 μmol/L 5‐aza‐dC was supplemented to the growth medium three times, every 24 h over a 72 h period. For IFN-γ treatment, melanoma cells were treated once with recombinant IFN-γ (1000 U/ml IFN-γ, PeproTech, Rocky Hill, NJ, USA) after 48 hours. Overall, we repeated the experiment in four independent experimental setups (Experiment 1-4). We conducted the first run of the experiment in six replicates (Experiment 1.1-1.6).

### 2.4 Methylation analysis

*HumanMethylation450 BeadChip analysis*: Gene methylation data generated by the TCGA Research Network were downloaded from the UCSC Xena browser (www.xena.ucsc.edu). Data on gene methylation (Infinium HumanMethylation450 BeadChip, Illumina, Inc., San Diego, CA, USA) from the TCGA Research Network were available from *N* = 470 patient samples. Methylation levels (*β*-values) were calculated: *β*-value = (Intensity_Methylated) / (Intensity_Methylated + Intensity_Unmethylated + α) [61]. The constant offset α was set to 0. *β*-values (values between 0 and 1) were multiplied with the factor 100% in order to estimate percent methylation (0 to 100%). HumanMethylation450 BeadChip data (*β*-values) from melanoma cell lines, melanocytes, and isolated immune cells were downloaded from GEO database (GSE51547, GSE95816, GSE44662, GSE86355, GSE87650, GSE71245, GSE59250).

**Fig. 1.**
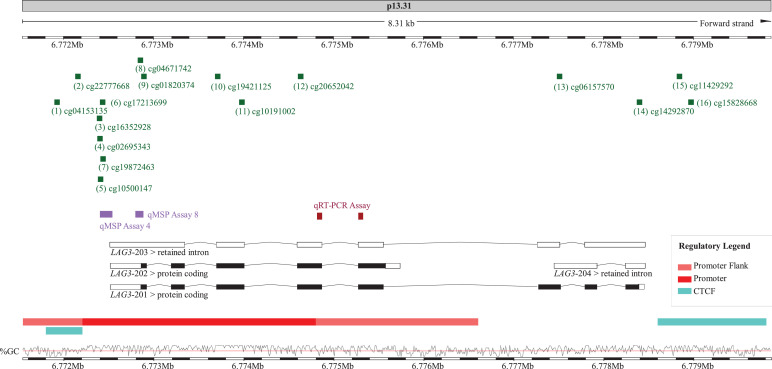
**Overview of analyzed methylation sites and genomic organization of *LAG3*.** Shown is chromosome 12, position 6,771,547-6,779,861, including the *LAG3* gene, its transcripts and regulatory elements (promoter, promoter flank, and CTCF binding sites), the investigated sequences (qMSP assays, HumanMethylation450 BeadChip beads, qRT-PCR assay). The target sites of the BeadChip beads (1-16), qMSP assays (four and eight), and the qRT-PCR assay are based on Genome Reference Consortium Human Build 38 patch release 13 (GRCh38.p13). The illustration (modified) was exported from www.ensemble.org (release 98) [89]. cg04153135 (1), cg22777668 (2), cg16352928 (3), cg02695343 (4), cg10500147 (5), cg17213699 (6), cg19872463 (7), cg04671742 (8), cg01820374 (9), cg19421125 (10), cg10191002 (11), cg20652042 (12), cg06157570 (13), cg14292870 (14), cg11429292 (15), cg15828668 (16).

*Quantitative methylation-specific real-time PCR (qMSP):* Furthermore, we used two qMSP assays (qMSP assays 4 and 8, Fig. 1), previously established and described by Klümper et al. [9] to quantify *LAG3* promoter methylation using bisulfite-converted template DNA. In brief, bisulfite DNA preparation from formalin-fixed and paraffin-embedded tissue (FFPET) specimens (UHB and ICB cohorts) was conducted after macrodissection of tumor tissues from sections mounted on glass slides. Tissue lysis and bisulfite conversion (FFPETs and cell line A375) was performed using the innuCONVERT Bisulfite All-In-One Kit (Analytik Jena, Jena, Germany) according the manufacturer's instructions. PCR reactions were performed in 20 µl volumes (buffer composition as previously described [62]) containing 20 ng bisulfite converted DNA (quantified via UV-VIS spectrophotometry) and 0.4 µM each primer and 0.2 µM each probe (qMSP assay 4 forward primer: aaccccctcaaactttccacta, reverse primer: gttttgttggtttttgggtttttatttt, probe_methylated_: 6-FAM-tagggtttacggtttcgtttcgt-BHQ-1, probe_unmethylated_: HEX-gtattttagggtttatggttttgttttgta-BHQ-1; qMSP assay 8 forward primer: ctttccttttctaacctcctttta, reverse primer: gtaagtttaggaattgagttttttatatt, probe_methylated_: 6-FAM-tggtttgggtagcgttgagttt-BHQ-1, probe_unmethylated_: HEX-atggtttgggtagtgttgagttttt-BHQ-1). qMSP was carried out using a 7900HT Fast Real-Time PCR system (Applied Biosystems, Waltham, MA, USA) with the following temperature profile: 20 min at 95°C and 40 cycles with 15 sec at 95°C, 2 sec at 62°C, and 60 sec at 52°C. We calculated percentage methylation levels using cycle threshold (CT) values obtained from probes specifically binding to bisulfite-converted methylated (CT_methylated_) and unmethylated (CT_unmethylated_) DNA, respectively (Methylation [%] = 100%/(1+2^CTmethylated–CTunmethylated^).

### 2.5 mRNA expression analysis

*RNA-Seq analysis:* mRNA data provided by the TCGA Research Network (http://cancergenome.nih.gov/) were generated by means of the Illumina HiSeq 2000 RNA Sequencing Version 2 analysis (Illumina, Inc., San Diego, CA, USA). Expression data of level 3 were downloaded from the TCGA webpage. Normalized counts (n.c.) per genes were calculated using the SeqWare framework via the RSEM algorithm [63]. mRNA expression levels were generated by means of HumanHT-12 V4.0 Gene Expression BeadChip (Illumina, Inc., San Diego, CA, USA). Raw data was downloaded from the GEO webpage.

*Quantitative reverse transcriptase PCR (qRT-PCR):* We quantified *LAG3* mRNA expression levels in 5-azacytidine and IFN-γ treated A375 melanoma cell lines my means of qRT-PCR. RNA extraction was performed using the NucleoSpin® RNA Kit (Machery-Nagel, Düren, Germany) according to the manufacturer's instructions. Complementary DNA (cDNA) was prepared using 500 ng of total RNA using the SuperScript™ III reverse transcriptase (ThermoFisher Scientific, Waltham, MA, USA) and oligo‐dT primers according to the manufacturer's instructions. Our qRT-PCR assay included an intron-spanning primer design and a probe targeting the splice site (*LAG3* forward primer: cctacagagatggcttcaacgtc, *LAG3* reverse primer: ggaacctgctccagcgtaca, *LAG3* probe: 6-FAM-ctcactgttctgggtctggagccc-BHQ-1). The primers amplify the mRNA sequence transcribed from the genomic region 12: 6,774,814-6,774,864 and 12: 6,775,273-6,775,319 (Fig. 1). The house keeping genes *ACTB* and *GAPDH* were used as references for normalization (*ACTB* forward primer: atgtggccgaggactttgatt, *ACTB* reverse primer: agtggggtggcttttaggatg, *ACTB* probe: 6-FAM-gaaatrmgtkgttacaggaagtccct-BHQ-1 [wobbles; r: a/g, m: a/c, k: g/t]; *GAPDH* forward primer: tgcaccaccaactgcttagc, *GAPDH* reverse primer: ggcatggactgtggtcatgag, *GAPDH* probe: 6-FAM-ctggccaaggtcatccatgacaact-BHQ-1). Buffer and cycling conditions were applied as used for the qMSP assays as described above with minor modifications (elongation temperatures: 58°C [*ACTB* and *GAPDH*] and 60°C [*LAG3*]; 0.16 µM each probe, 0.4 µM each *LAG3* primer, 0.2 µM each *ACTB* and *GAPDH* primer). One µl cDNA template per 20 µl PCR was analyzed. Relative *LAG3* expression levels were calculated using the ΔCT method.

### 2.6 Statistics

Statistical analyses were conducted using SPSS, version 23.0 (SPSS Inc., Chicago, IL, USA). Correlations were calculated using Spearman's rank correlation (Spearman's *ρ*). Mean value comparisons were performed with Wilcoxon-Mann-Whitney *U* (two groups), Kruskal-Wallis (>2 groups) test, and paired *t*-tests. Multiple comparisons between groups were further tested with one-way ANOVA and post-hoc Bonferroni test. Survival analyses were performed using the Kaplan-Meier method and Cox proportional hazards regression. Overall survival was censored after 5 years (1,825 days) in order to reduce the influence of age-related deaths. *P*-values refer to log-rank and Wald tests, respectively. For Kaplan-Meier analysis methylation levels and mRNA expression levels were dichotomized based on an optimized cut-off. Cox proportional hazards analyses were performed with continuous methylation and mRNA expression data (with and without taking the logarithm to the base of 2). Two-sided *P*-values <0.05 were considered statistically significant.

### 2.7 Role of funders

The funders had no role in study design, data collection and analysis, interpretation, decision to publish, or preparation of the manuscript; or any aspect pertinent to the study.

## 3 Results

### 3.1 Association of *LAG3* mRNA expression and methylation

Transcriptional gene silencing is often conferred by promoter methylation. To test the hypothesis that *LAG3* expression is controlled by DNA methylation, we correlated the methylation levels of 16 CpG sites within the *LAG3* gene with RNA-Seq expression data of *N* = 468 melanoma samples from the TCGA. To analyze methylation of the *LAG3* gene we made use of the Infinium HumanMethylation450 BeadChip. Fig. 1 illustrates the genomic organization of the *LAG3* gene with its two protein coding transcripts, *LAG3*-201 and *LAG3*-202, sharing the same transcription start site. In the region of the transcription start site the localization of an extended promoter and its flank is predicted. CpG sites three to 12 are located in the promoter. CpG sites probed by beads one and two are located in an upstream promoter flank, including a promoter-embedded CTCF binding site. CpG sites 13 and 14 are located within the gene body. The CpGs 15 and 16 are situated in a downstream CTCF binding site.

**Fig. 2:**
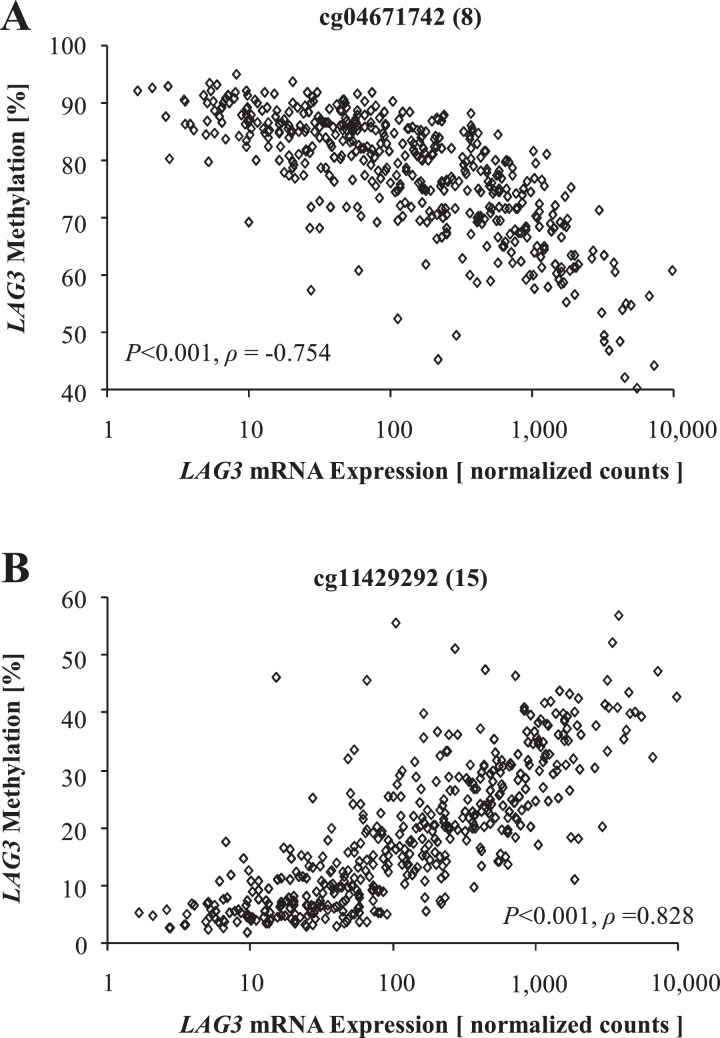
**Correlation of *LAG3* methylation and mRNA expression in *N* = 468 melanoma samples.***LAG3* methylation of two loci targeted by HumanMethylation450 BeadChip beads cg04671742 (8) and cg11429292 (15) are shown.

We found significant inverse correlations between *LAG3* methylation and gene expression in 12 out of 16 analyzed beads (Table 1). Inverse correlation between methylation levels and mRNA expression in melanoma tissue is exemplarily shown for the CpG site targeted by bead eight (Fig. 2A), where correlation was most pronounced. Interestingly, 11 CpG sites showing a significant inverse correlation between *LAG3* methylation and gene expression were located in the promoter and upstream promoter flank (beads one to 11). On the contrary, when we analyzed bead 14 located in the gene body and bead 15 that binds in the CTCF binding site, we found a significant positive correlation. This is exemplified in Fig. 2B for bead 15 where this correlation was highly significant.

**Table 1:** **Correlations of *LAG3* methylation with *LAG3* mRNA expression, lymphocyte score, and overall survival.***LAG3* methylation was determined at 16 different loci targeted by HumanMethylation450 BeadChip beads (Fig. 1). Significant data are shown in boldface.

Analyte	Bead no.	Correlation with *LAG3* mRNA expression		Correlation with lymphocyte score		Overall survival	
		Spearman's*ρ*	*P*-value	Spearman's *ρ*	*P*-value	Hazard ratio [95% CI]	*P*-value
*LAG3* mRNA	NA	NA	NA	**0.503**	**<0.001**	**0.86 [0.79-0.93]**	**<0.001**
cg04153135	1	**-0.384**	**<0.001**	**-0.335**	**<0.001**	4.25 [0.33-54.0]	0.27
cg22777668	2	**-0.520**	**<0.001**	**-0.380**	**<0.001**	1.18 [0.69-2.01]	0.55
cg16352928	3	**-0.612**	**<0.001**	**-0.422**	**<0.001**	1.53 [0.95-2.46]	0.079
cg02695343	4	**-0.644**	**<0.001**	**-0.465**	**<0.001**	1.63 [0.99-2.69]	0.055
cg10500147	5	**-0.614**	**<0.001**	**-0.450**	**<0.001**	1.27 [0.94-1.70]	0.12
cg17213699	6	**-0.670**	**<0.001**	**-0.497**	**<0.001**	1.60 [0.98-2.61]	0.058
cg19872463	7	**-0.663**	**<0.001**	**-0.483**	**<0.001**	1.41 [0.92-2.15]	0.12
cg04671742	8	**-0.754**	**<0.001**	**-0.477**	**<0.001**	2.45 [0.88-6.86]	0.087
cg01820374	9	**-0.522**	**<0.001**	**-0.404**	**<0.001**	1.18 [0.77-1.82]	0.45
cg19421125	10	**-0.673**	**<0.001**	**-0.498**	**<0.001**	1.39 [0.85-2.27]	0.19
cg10191002	11	**-0.667**	**<0.001**	**-0.511**	**<0.001**	**1.97 [1.05-3.68]**	**0.035**
cg20652042	12	**-0.713**	**<0.001**	**-0.481**	**<0.001**	**3.25 [1.07-9.88]**	**0.037**
cg06157570	13	**-0.120**	**0.005**	**-0.183**	**0.001**	0.59 [0.25-1.37]	0.22
cg14292870	14	**0.440**	**<0.001**	**0.320**	**<0.001**	**0.71 [0.53-0.94]**	**0.017**
cg11429292	15	**0.828**	**<0.001**	**0.472**	**<0.001**	**0.70 [0.59-0.83]**	**<0.001**
cg15828668	16	0.054	0.25	0.096	0.082	1.42 [0.83-2.46]	0.20

NA: Not Applicable

### 3.2 Correlation of *LAG3* methylation and mRNA expression with immune cell infiltrates

**Fig. 3:**
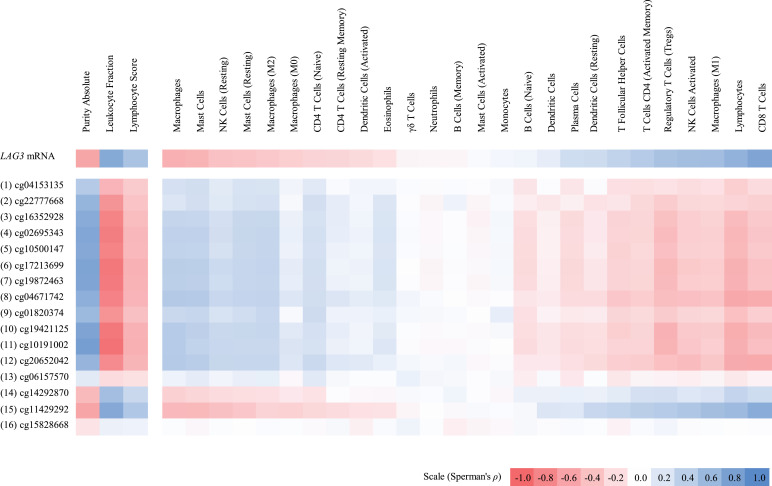
**Correlation of *LAG3* methylation and mRNA expression with immune cell infiltrates.** Shown are Spearman's *ρ* correlation coefficients between *LAG3* methylation and mRNA expression with leukocyte fraction (mRNA: *N* = 468; methylation: *N* = 470) and distinct immune cell infiltrate signatures (mRNA: *N* = 468; methylation: *N* = 469). Immune cell infiltrates include RNA signatures of lymphocytes (including naive B cells, memory B cells, naive CD4^+^ T cells, activated and resting CD4^+^ memory T cells, T follicular helper cells, regulatory T cells, CD8^+^ T cells, γδ T cells, activated and resting NK cells, and plasma cells), macrophages (including monocytes and M0/M1/M2 macrophages), dendritic cells (including resting and activated dendritic cells), mast cells (including activated and resting mast cells), CD4^+^ T cells (including naive, activated memory, and resting memory CD4^+^ T cells), eosinophils, and neutrophils. *P*-values and Spearman's *ρ* correlation coefficients can be found in Supplemental Table 1.

Considering the function of LAG3 as an inhibitory checkpoint receptor known to be expressed on leukocytes, we assumed expression of *LAG3* to correlate with levels of tumor infiltrating immune cells. Following up on this assumption, we investigated positive and negative correlations of *LAG3* methylation and mRNA expression with lymphocyte score and leukocyte fraction in the TCGA cohort. As expected, we found a significant positive correlation between *LAG3* mRNA expression with lymphocyte score and leukocyte fraction (Table 1, Fig. 3). Accordingly, at the CpG sites targeted by beads one to 13, mainly located in the promoter region, lymphocyte score and leukocyte fraction showed a significant inverse correlation with *LAG3* methylation. We further investigated the TCGA data with regard to the tumor content of the samples. Here, *LAG3* mRNA expression and methylation patterns were opposed to the pattern observed in the TILs: Purity of tumor tissue and tumor cell content (% nuclei that are tumor cells) showed significant inverse correlations with *LAG3* mRNA expression. Accordingly, purity of tumor tissue and tumor cell content significantly correlated with *LAG3* methylation of the CpGs targeted by beads one to 13 (purity of tumor tissue) and of the CpGs targeted by beads one to 12 (Fig. 3, Supplemental Table 1). For bead 14, that targets a CpG site located within the gene body and bead 15 probing a CpG within the CTCF binding site, an inverse correlation between *LAG3* methylation and tumor cell content/purity of the tumor tissue could be shown. Concordantly, we detected significant positive correlations of *LAG3* methylation with leukocyte fraction and lymphocyte score. Taken together, we demonstrated a correlation between hypomethylation in the regions targeted by beads one to 13 and an increased *LAG3* mRNA expression. Hypermethylation in the regions targeted by beads one to 12 was correlated with tumor cell content and purity, whereas hypermethylation in the regions for bead 14 and 15 correlated with lymphocyte score and leukocyte fraction.

To follow up on our results based on the TCGA data we set up a validation cohort composed of *N* = 120 melanoma samples (UHB cohort). We used a qMSP assay targeting the CpG sites eight and four (Fig. 1), which had shown highly significant correlations in the analysis of the TCGA cohort. We correlated methylation levels with lymphocyte score, evaluated by histopathologic examination. Mean lymphocyte score was 1.18 [95% CI, 0.93-1.43], mean percentage leukocytes in the tumor made up 5.89% [95% CI, 4.68%-7.09%], mean percentage of tumor cells were 93.56% [95% CI, 92.31%-94.81%]. Valid results for methylation and histopathologic analysis were available for *N* = 114 (qMSP assay 4) and *N* = 117 (qMSP assay 8) melanoma samples, respectively. We observed a significant inverse correlation between methylation of the CpG site targeted by bead eight (qMSP assay 8) and lymphocyte score (*ρ*=-0.405, *P* < 0.001) and the leukocyte fraction (*ρ*=-0.339, *P* < 0.001). Accordingly, we demonstrated a positive correlation between methylation of CpG site eight with tumor cell fraction (*ρ*=0.308, *P* = 0.011). We observed a significant inverse correlation between methylation of the CpG site four (qMSP assay 4) and lymphocyte score (*ρ*=-0.238, *P* < 0.001) and the leukocyte fraction (*ρ*=-0.249, *P* = 0.008). We found a positive correlation between methylation of CpG site eight with tumor fraction (*ρ*=0.268, *P* = 0.004). Accordingly, our data confirmed the results obtained from the TCGA data.

### 3.3 Correlation of *LAG3*methylation and mRNA expression with immune cell subsets

Tumor tissue is composed of different compounds, including tumor cells, stroma, and distinct immune cell subsets. We correlated RNA-Seq signatures of B cells, CD4^+^ and CD8^+^ T cells, neutrophils, macrophages, and dendritic cells with *LAG3* methylation levels (Figure 3). Our preliminary results had shown an association of *LAG3* promoter hypomethylation with increased levels of tumor infiltrating immune cells, whereas infiltration of immune cells was correlated with hypermethylation of the downstream CTCF binding site. Analyses of the single immune cell subsets demonstrated significant correlations between *LAG3* promoter hypomethylation and *LAG3* mRNA levels with proinflammatory and activated immune cell subsets, particularly with activated NK cells, CD8^+^ T cells, and activated CD4^+^ memory cells, which is in line with the published knowledge of LAG3 expression on activated immune cells [13,64]. *LAG3* promoter hypomethylation and mRNA expression showed a correlation with an RNA-Seq signature of Tregs. Accordingly, Tregs have been shown to express LAG3 in dynamic levels, depending on the state of activation, and with high levels described in melanoma. We observed a positive correlation of *LAG3* mRNA expression and promoter hypomethylation and RNA-Seq signatures of proinflammatory M1 macrophages and resting DCs. *LAG3* promoter hypermethylation correlated with RNA-Seq signatures of resting NK cells, naïve CD4^+^ T cells, M0 and anti-inflammatory M2 macrophages, and activated DCs. This finding is of interest as little is known about the role of LAG3 for plasmacytoid DCs. In line with our previous results, hypermethylation of the CTCF binding site targeted by CpGs 15 and 16 showed an opposite pattern and was associated with RNA-Seq signatures of proinflammatory or activated leukocyte subsets, whereas hypomethylation of the CTCF binding site correlated with RNA-Seq signatures of the anti-inflammatory, undifferentiated, and mainly resting immune cell subsets (Fig. 3).

### 3.4 *LAG3* methylation in cell subsets from peripheral blood, melanocytes, and melanoma cell lines

**Fig. 4.**
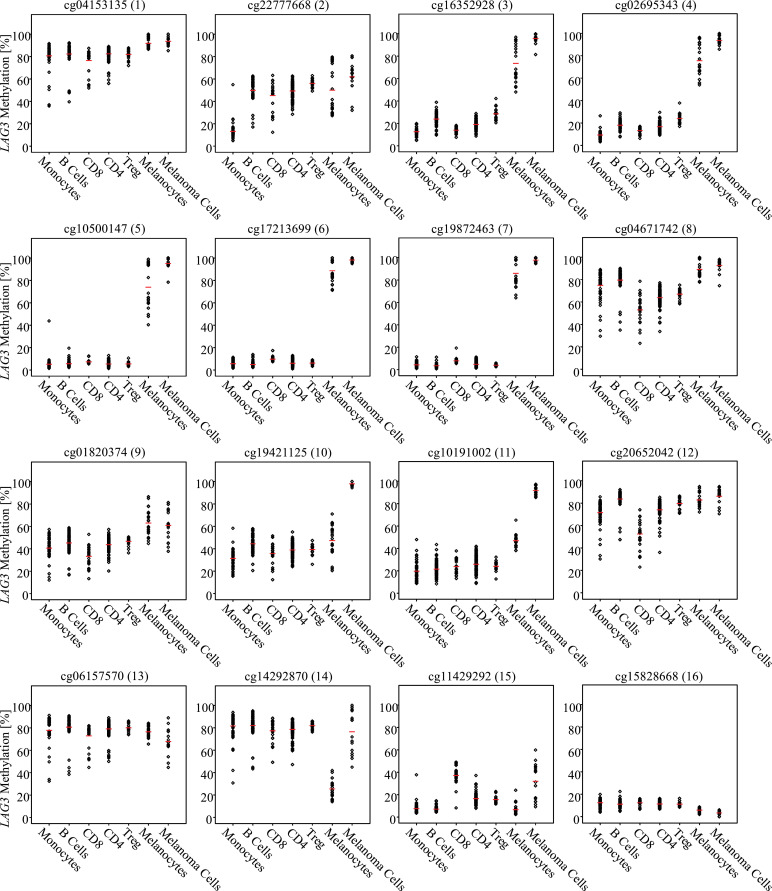
***LAG3* methylation in leucocytes, melanocytes, and melanoma cell lines.***LAG3* methylation at 16 sites in isolated leucocytes (*N* = 53 monocytes, *N* = 60 B cells, *N* = 24 CD8^+^ T cells, *N* = 97 CD4^+^ T cells, and *N* = 18 regulatory T cells) from healthy donors, melanocytes (*N* = 23) and melanoma cell lines (*N* = 16). ANOVA *P* < 0.001 for all 16 sites. Results from pairwise Bonferroni post-hoc comparisons are listed in Supplemental Table 2.

In a next step, we investigated *LAG3* methylation in melanocytes and melanoma cell lines and in isolated peripheral blood mononuclear cells (PBMCs), comprising lymphocytes and monocytes. In line with our previous results demonstrating significant correlations between *LAG3* mRNA expression and lymphocyte score and inverse correlations between *LAG3* promoter methylation and lymphocyte score in whole tumor tissue (Table 1), isolated PBMCs showed pronounced *LAG3* hypomethylation in the CpG sites three to seven and 11, all of them located in the promoter region (Fig. 4). Melanoma cell lines, however, showed hypermethylation in the *LAG3* promoter region. This result is in line with the correlations demonstrated in the TCGA data analysis, showing that hypermethylation in the regions targeted by beads one to 12 was correlated with tumor cell content and purity. Methylation pattern in melanocytes resembled the pattern observed in melanoma cells and showed high levels of methylation in the CpG sites one to nine. However, the variability observed in the methylation pattern was larger. CpG sites 11, 14, and 15 showed lower methylation levels compared with melanoma cells. The CpG site 16 showed only a trend toward a correlation between lymphocyte score and *LAG3* methylation. Accordingly, there were only marginal but significant differences between methylation in melanoma cells and immune cells (Fig. 4).

### 3.5 Correlation of *LAG3* methylation and interferon-γ signature

With regards to the function of LAG3 as an inhibitory receptor involved in immune response, we analyzed the association of mRNA expression and methylation of *LAG3* with an IFN-γ signature defined by the mRNA expression of IFN-γ (*IFNG*) and IFN-γ–regulated genes (*STAT1, STAT2, JAK2*, and *IRF9*; Table 2). As expected *LAG3* mRNA expression was significantly correlated with an INF-γ signature. In line with our previous findings, *LAG3* methylation in the promoter regions showed an inverse correlation with an INF-γ signature, whereas significant positive correlations were demonstrated in the CpG target beads 14 and 15, located in the gene body and the CTCF binding site.

**Table 2:** **Correlations of *LAG3* methylation and mRNA expression with IFN-γ-signature**. Correlations of *LAG3* methylation and mRNA expression with IFN-γ (*IFNG*) and IFN-γ–regulated genes (*STAT1, STAT2, JAK2*, and *IRF9*). DNA methylation was determined at sixteen different loci targeted by HumanMethylation450 BeadChip beads (Fig. 1). Significant data are shown in boldface. Data were obtained from *N* = 468 tumor samples, respectively.

Analyte	Bead no.	*IFNG*		*STAT1*		*STAT2*		*JAK2*		IRF9	
		Spearman's *ρ*	*P*-value	Spearman's *ρ*	*P*-value	Spearman's *ρ*	*P*-value	Spearman's *ρ*	*P*-value	Spearman's ρ	P-Value
*LAG3* mRNA	NA	**0.90**	**<0.001**	**0.74**	**<0.001**	**0.40**	**<0.001**	**0.44**	**<0.001**	**0.66**	**<0.001**
cg04153135	1	**-0.40**	**<0.001**	**-0.28**	**<0.001**	-0.07	0.15	**-0.21**	**<0.001**	**-0.24**	**<0.001**
cg22777668	2	**-0.52**	**<0.001**	**-0.39**	**<0.001**	-0.09	0.055	**-0.28**	**<0.001**	**-0.32**	**<0.001**
cg16352928	3	**-0.60**	**<0.001**	**-0.45**	**<0.001**	**-0.21**	**<0.001**	**-0.31**	**<0.001**	**-0.38**	**<0.001**
cg02695343	4	**-0.62**	**<0.001**	**-0.47**	**<0.001**	**-0.21**	**<0.001**	**-0.33**	**<0.001**	**-0.40**	**<0.001**
cg10500147	5	**-0.59**	**<0.001**	**-0.44**	**<0.001**	**-0.18**	**<0.001**	**-0.32**	**<0.001**	**-0.37**	**<0.001**
cg17213699	6	**-0.65**	**<0.001**	**-0.49**	**<0.001**	**-0.24**	**<0.001**	**-0.35**	**<0.001**	**-0.43**	**<0.001**
cg19872463	7	**-0.64**	**<0.001**	**-0.48**	**<0.001**	**-0.23**	**<0.001**	**-0.34**	**<0.001**	**-0.42**	**<0.001**
cg04671742	8	**-0.74**	**<0.001**	**-0.58**	**<0.001**	**-0.33**	**<0.001**	**-0.36**	**<0.001**	**-0.45**	**<0.001**
cg01820374	9	**-0.52**	**<0.001**	**-0.38**	**<0.001**	**-0.16**	**<0.001**	**-0.23**	**<0.001**	**-0.33**	**<0.001**
cg19421125	10	**-0.65**	**<0.001**	**-0.52**	**<0.001**	**-0.33**	**<0.001**	**-0.30**	**<0.001**	**-0.50**	**<0.001**
cg10191002	11	**-0.65**	**<0.001**	**-0.50**	**<0.001**	**-0.30**	**<0.001**	**-0.33**	**<0.001**	**-0.48**	**<0.001**
cg20652042	12	**-0.70**	**<0.001**	**-0.56**	**<0.001**	**-0.31**	**<0.001**	**-0.32**	**<0.001**	**-0.45**	**<0.001**
cg06157570	13	**-0.15**	**<0.001**	-0.09	0.055	0.03	0.49	-0.02	0.61	-0.02	0.62
cg14292870	14	**0.45**	**<0.001**	**0.42**	**<0.001**	**0.21**	**<0.001**	**0.33**	**<0.001**	**0.33**	**<0.001**
cg11429292	15	**0.84**	**<0.001**	**0.70**	**<0.001**	**0.34**	**<0.001**	**0.46**	**<0.001**	**0.54**	**<0.001**
cg15828668	16	0.04	0.45	**0.11**	**0.016**	0.01	0.76	0.07	0.14	0.04	0.34

NA: Not Applicable

### 3.6 Tumor cell-intrinsic *LAG3* mRNA expression in dependence on pharmacological demethylation and IFN-γ stimulation

**Fig. 5.**
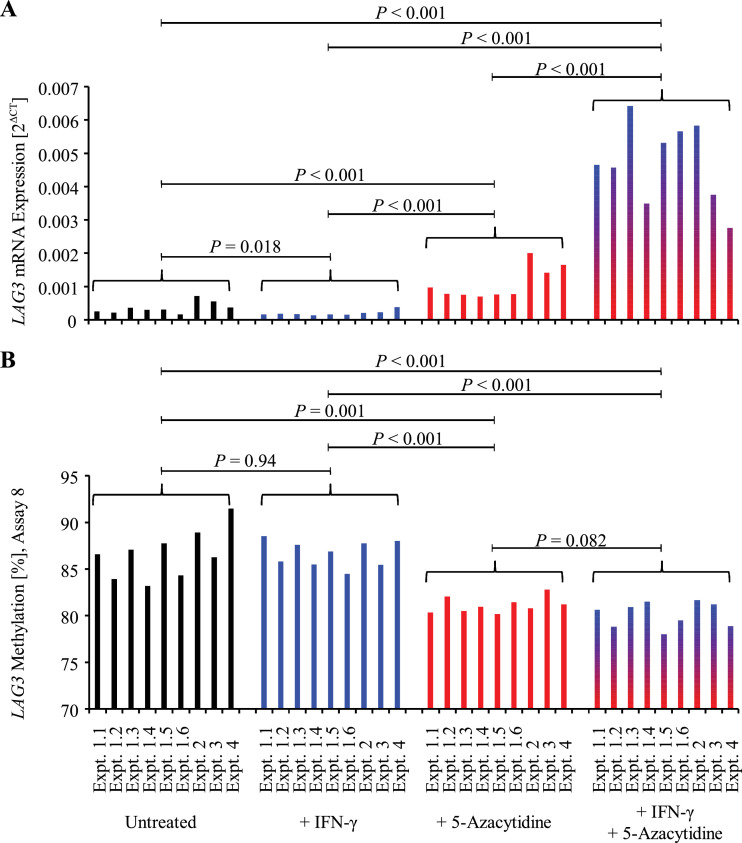
**Inducibility of tumor cell-intrinsic *LAG3* mRNA expression by pharmacological demethylation and IFN-γ stimulation.** (**A**) Treatment of human melanoma cell line A375 *in vitro* with hypomethylating agent 5‐azacitidine leads to increased *LAG3* expression. Combined 5‐azacitidine treatment and IFN-γ stimulation leads to further increase of *LAG3* expression. IFN-γ stimulation alone resulted in a decreased *LAG3* expression. (**B**) Treatment of human melanoma cell line A375 *in vitro* with hypomethylating agent 5‐azacitidine leads to decreased *LAG3* methylation levels. *P*-values refer to paired *t*-tests.

Knowledge on tumor cell-intrinsic mRNA expression of *LAG3* in melanoma and its transcriptomic regulation is scarce. We therefore investigated *LAG3* mRNA expression and the influence of the hypomethylating agent 5-azacytidine on *LAG3* in the melanoma cell line A375. The experiment included four different treatment arms: 1. no treatment, 2. IFN- γ, 3. 5-azacytidine, 4. IFN- γ and 5-azacytidine (Fig. 5A and Fig. 5B). The experiment was performed nine times (six replicates within experiment one (expt. 1.1-1.6) and three independent experiments (expt. 2-4)). We quantified promoter methylation levels at the promoter CpG site targeted by bead eight using qMSP. As expected, methylation levels in 5-azacytidine treated cell lines were significantly lower than in the groups without 5-azacytidine treatment (Fig. 5B). IFN-γ treatment showed no significant effects on methylation levels. In a next step, we quantified *LAG3* mRNA levels by means of qRT-PCR. Contrary to our expectations, IFN-γ stimulation alone in the control group without 5-azacytidine led to a significant decrease of *LAG3* mRNA expression (Fig. 5A). 5-azacytidine treatment, however, resulted in a significant increase in *LAG3* expression. Interestingly, IFN-γ stimulation in addition to 5-azacytidine treatment led to a significant and sharp increase of *LAG3* mRNA expression.

### 3.7 Associations of *LAG3* methylation and mRNA expression with clinicopathological parameters and molecular features

We analyzed possible associations of *LAG3* methylation patterns and mRNA expression with clinicopathological, epidemiologic, and molecular features based on data of the TCGA cohort to identify prognostically significant parameters. A detailed analysis of associations and correlations of *LAG3* methylation and mRNA expression is summarized in Supplemental Table 1. We did not find significant correlations of *LAG3* mRNA expression or methylation with age or gender. Analysis of *LAG3* mRNA expression in different tumor tissue sites showed higher expression of *LAG3* mRNA in regional lymph nodes and cutaneous metastases compared to primary tumors and distant metastases. *LAG3* mRNA expression was lowest in distant metastases. These differences in expression may also be credited to different tumor cell content. Accordingly, we found differences in the methylation status of *LAG3* depending on the tumor site. Mean promoter methylation in CpG sites targeted by beads two to 12 were lowest in tissue obtained from lymph nodes. Methylation levels in the CpG sites targeted by beads 14 to 16, including the CTCF binding site of the *LAG3* gene, were highest in lymph node and cutaneous metastases, a finding that may be attributed to an increased lymphocyte infiltration. We refrained from conducting further subgroup analysis to compare *LAG3* methylation in visceral metastases of different tumor sites as the single subgroups included in the TCGA data are too small to allow for valid subgroup analyses.

### 3.8 Prognostic value of *LAG3* methylation and mRNA expression for melanoma survival

**Fig. 6:**
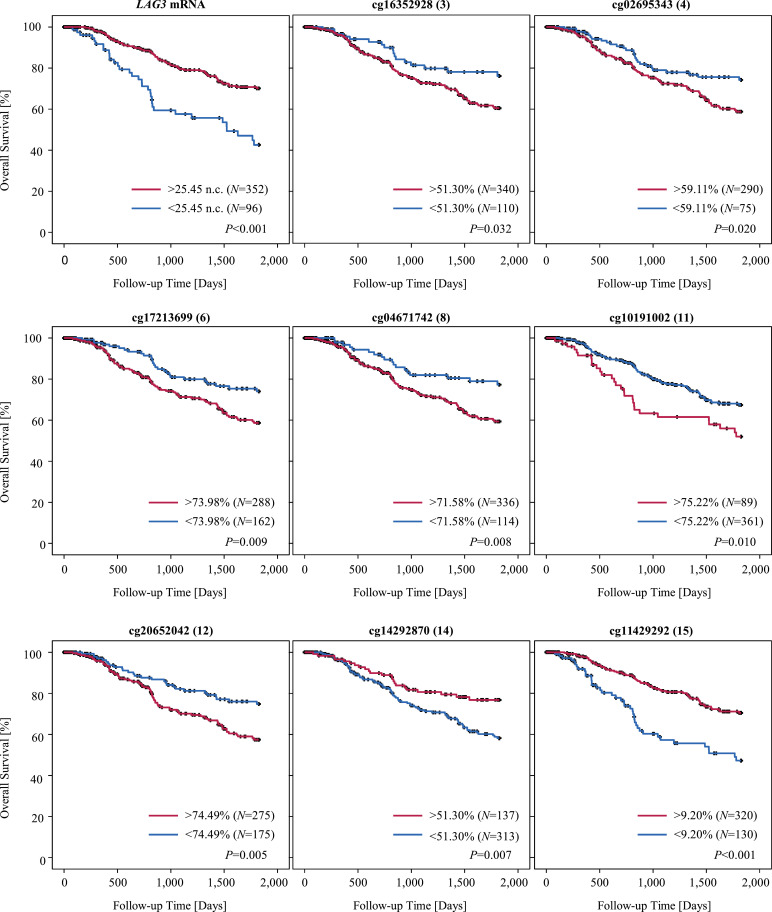
**Kaplan-Meier analysis of overall survival in melanoma patients stratified according to *LAG3* methylation and mRNA expression.** Patient samples were dichotomized based on optimized cutoffs.

We tested the prognostic significance of *LAG3* mRNA expression and methylation in the TCGA cohort. We tested methylation and mRNA expression levels as continuous variates to avoid overfitted models due to the introduction of cutoffs for patient sample classification. We could demonstrate that increased *LAG3* mRNA expression was associated with a significantly improved overall survival of melanoma patients (Hazard ratio [HR] = 0.86 [95% CI: 0.79-0.93]; *P* < 0,001, Wald test; Table 1). In accordance with this result, we found that hypermethylation of CpG sites targeted by 13 out of 16 analyzed beads was associated with a poorer outcome, the results reached statistical significance in four beads under investigation (Table 1). When we further dichotomized the mRNA expression and methylation data for optimized cutoffs, Kaplan-Meier survival curves confirmed a better survival outcome for patients with higher *LAG3* mRNA expression (Fig. 6). Exemplary, Kaplan-Meier survival graphs for methylation status in CpG sites in the promoter region of the gene (beads 3, 4, 6, 8, 11, 12) underlined our findings that *LAG3* promoter hypomethylation correlated with a better survival outcome. In contrast, when we characterized methylation of CpG sites in the gene body and the CTCF binding site of the gene targeted by beads 14 and 15, we found that hypermethylation of these CpG sites correlated with a better survival outcome of melanoma patients (Fig. 6).

### 3.9 Association of *LAG3* methylation with progression-free survival in melanoma patients treated with immune checkpoint blockade

**Fig. 7.**
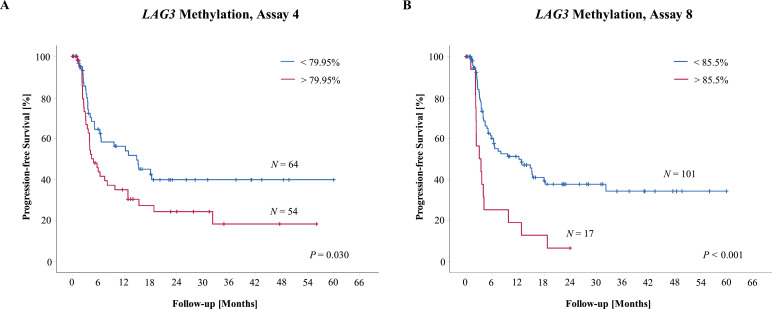
**Kaplan-Meier analysis of progression-free survival in *N* = 118 metastatic melanoma patients treated with ICB and stratified according to *LAG3* methylation.** Patient samples were dichotomized based on optimized cutoffs. (**A**) Kaplan-Meier analysis stratified according to *LAG3* methylation quantified by assay four. (**B**) Kaplan-Meier analysis stratified according to *LAG3* methylation quantified by assay eight.

We further tested *LAG3* methylation as a predictive biomarker for disease progression in metastatic melanoma patients with ICB (*N* = 118). We correlated progression-free survival (PFS) with methylation levels in pre-treatment samples measured by qPCR targeting the CpG sites eight and four. In an univariate Cox proportional analysis we found a trend towards a shorter PFS in patients with *LAG3* hypermethylated tumors (CpG site 8: HR = 1.03 [95% CI: 0.99-1.06]; *P* = 0.063; CpG site 4: HR = 1.04 [95% CI: 0.99-1.03]; *P* = 0.061; Wald test). When we further dichotomized the methylation data applying optimized cutoffs, Kaplan-Meier survival curves demonstrated a better PFS for melanoma patients with hypomethylated tumors (Fig. 7).

## 4 Discussion

Immunotherapy of advanced melanoma with PD-1 and PD-L1 blocking antibodies as well as CTLA-4 antibodies has become state of the art in the clinics alongside targeted therapies. However, resistance mechanisms against immune checkpoint blockade pose a major challenge in the clinical management of advanced melanoma, calling for a rational combination targeting of different immune checkpoints [65]. LAG3 has been shown to play a crucial role in the negative regulation of T cells under physiological conditions and, importantly, in tumor cell - immune cell interactions [10], thus making it an attractive additional target in immunotherapy. In view of the thriving landscape of immunotherapeutic molecules, biomarkers that help to stratify patients for the most suitable therapy are urgently needed.

In this study we investigate the epigenetic regulation of *LAG3* expression via DNA methylation in melanoma and evaluate the feasibility of *LAG3* methylation as an epigenetic biomarker correlating *LAG3* mRNA expression, immune cell infiltration, molecular and clinicopathological characteristics, overall and progression-free survival. Our results strongly suggest an epigenetic regulation of *LAG3* via DNA methylation. We found significant correlations between *LAG3* methylation and mRNA expression with lymphocyte score, signatures of tumor infiltrating lymphocytes, IFN-γ signature, and survival, suggesting a prognostic and predictive significance of *LAG3* in melanoma. In addition, we demonstrated that *LAG3* mRNA is expressed at low levels in a melanoma cell line and could be induced by demethylating agents, thus confirming a transcriptional regulation of *LAG3 via* DNA methylation.

We demonstrated significant inverse correlations of *LAG3* mRNA expression with methylation of CpG sites in 12 out of 16 analyzed beads in the TCGA malignant melanoma cohort that were mainly located in the promoter region of the *LAG3* gene. On the contrary, there was a positive correlation of methylation and mRNA expression at two sites located in the gene body and within the binding site of the transcriptional repressor CTCF. The results strongly support the notion of an epigenetic regulation of *LAG3* expression *via* DNA methylation. Different functional effects of methylation within distinct genomic regions may point towards a different regulation by variable enhancer elements. Thus, the transcriptional repressor CTCF is known to be involved in diverse regulatory functions, including transcriptional activation and repression, and plays a pivotal role in the organization of chromatin architecture [66].

LAG3 is expressed on activated CD4^+^ and CD8^+^ T cells, NK cells, B cells, DCs but also on Tregs and exhausted effector T cells [13,64]. In its physiologic function as immune checkpoint receptor, LAG3 signaling downregulates proliferation, activation and allows for homeostasis of T cells. In the state of chronic inflammation, LAG3 is expressed on dysfunctional CD8^+^ T cells [67] and is known to mediate immunosuppressive functions of Tregs [8], thereby signaling a state of T cell exhaustion in co-expression with other inhibitory molecules [2]. Our analysis revealed correlations of *LAG3* methylation and mRNA expression with distinct pro- and anti-inflammatory immune cell subsets in different stages of activation. Taken together we found correlations of *LAG3* promoter hypomethylation and *LAG3* mRNA expression with proinflammatory and activated immune cell subsets, particularly with activated NK cells, CD8^+^ T cells, and activated CD4^+^ T cells, which is in line with the published knowledge of LAG3 expression on activated immune cells [13,64]. Our analysis confirmed the associations of LAG3 with Tregs and showed that *LAG3* promoter hypomethylation and *LAG3* mRNA expression is correlated with an RNA-Seq signature of Tregs. Our results are in accordance with data of several tumor models which demonstrated Tregs to express LAG3 in dynamic levels, depending on the state of activation. High levels of LAG3 have been described on immunosuppressive Tregs in cancer [7] and specifically in melanoma [8]. We observed a positive correlation of *LAG3* promoter hypomethylation and *LAG3* mRNA expression with proinflammatory M1 macrophages. This is in line with a recent study describing *LAG3* as a so far unexplored gene marker within the molecular signature of a proinflammatory human macrophage subtype, which was challenged with IFN-γ plus lipopolysaccharides and TNFα [68]. The observed correlations between *LAG3* promoter methylation pattern and *LAG3* expression with DC infiltrates is of particular interest, as the influence of LAG3 on plasmacytoid DCs has not completely been unraveled, yet. Published data suggested that LAG3 plays a role in plasmacytoid DC biology and demonstrated LAG3 to be constitutively expressed on plasmacytoid DCs. The authors found that activated but not resting plasmacytoid DCs generated a substantial amount of sLAG3 [4]. The observed correlations between promoter hypomethylation and *LAG3* expression with resting DC infiltrates display the complex role of DCs in cancer immunology. LAG3 expression was also found on melanoma infiltrating plasmacytoid DCs which contributed to an immunosuppressive microenvironment [69]. Moreover, LAG3 expression in conjunction with PD-L1 expression has been demonstrated on tumor infiltrating CD4^+^ and CD8^+^ T cells and during the course of tumor cell - immune cell interaction [10]. The demonstrated correlations support the biologic significance of our results.

Melanoma tissue is composed of diverse cell types. We performed an independent comparison of *LAG3* methylation in melanocytes, melanoma cell lines, and isolated leukocyte subsets from peripheral blood. We observed differential methylation pattern and levels within melanocytes compared to melanoma cell lines at a significant number of CpG sites under investigation, which is of particular interest given the close relationship of these cell types springing from the same mesenchymal origin. Methylation analysis in melanoma cell lines and isolated leukocyte subsets (monocytes, B cells, CD8^+^ T cells, CD4^+^ T cells, and regulatory T cells) revealed striking differences in methylation patterns. Six CpG sites located in the promoter region showed pronounced hypomethylation in leukocytes compared to melanoma cell lines. Methylation data provided by the TCGA reflects this finding in whole tumor tissue including immune cells, melanoma cells, and stroma. We therefore assume, that hypomethylation of PBMCs corresponds to hypomethylation of tumor infiltrating lymphocytes in tumor tissues and may therefore serve as a surrogate biomarker for tumor infiltrating lymphocytes. However, this hypothesis needs further validation, in particular as analysis of PBMCs is limited by heterogeneity and PBMCs are derived from healthy donors and might differ from immune cells in cancer patients. Furthermore, the role of *LAG3* methylation in melanoma cells needs further evaluation. Recently, *LAG3* expression in clear cell renal carcinoma cell lines and a tumor cell-intrinsic LAG3 protein expression was reported [9]. However, knowledge of tumor cell-intrinsic expression of the LAG3 protein in melanoma is scarce. In a previous study no tumor cell-intrinsic LAG3 protein expression was detected in brain metastases from melanoma patients [70]. However, we confirmed a tumor cell-intrinsic and inducible mRNA expression of *LAG3* and a regulative role of *LAG3* DNA methylation in the melanoma cell line A375. Hypomethylation induced by 5-azacytidine led to a significant increase in *LAG3* mRNA expression, thereby strongly supporting our assumption of a transcriptional role of DNA methylation. According to our results, a recent study demonstrated the significance of *PD-L1* methylation in relation to *PD-L1* mRNA expression [43]. Whereas the role of LAG3 protein expression in immune cells, particularly within the orchestra of tumor microenvironment, has been studied in detail, the role of *LAG3* in cancer cells is not well understood. A comprehensive study across more than 1,100 samples of the Cancer Cell Line Encyclopedia showed that nearly 90% of T cell coinhibitory/costimulatory genes, among them *LAG3*, were not expressed or had low expression across the included cancer cell lines [71]. The authors concluded that the tumor cell-intrinsic role of immune checkpoints should also be considered when assessing the efficacy of anti-tumor immunotherapy [71].

Infiltration of melanomas with lymphocytes is commonly associated with a better prognosis and there is evidence that it may also predict a better response to immune checkpoint inhibitors [72]. We therefore investigated correlations between methylation status and leukocyte infiltration in the TCGA cohort. We found inverse correlations between lymphocyte score and *LAG3* methylation for most of the targeted CpG sites in the promoter of *LAG3* and a positive correlation for methylation and immune infiltrates at the CpG sites within the CTCF binding site. Analysis of our UHB validation cohort confirmed inverse correlations between *LAG3* methylation and leukocyte score in the promoter region. LAG3 expression is induced upon immune cell infiltration in tumor tissue [6]. Infiltration of CD8^+^ T cells is associated with an activation of the IFN-γ pathways [73,74]. Within a proinflammatory tumor microenvironment an activation-induced IFN-γ signature promotes the generation of MHC-II complexes [75] and LAG3 recognition of stable peptide-MHC-II complexes is critical for activity [76], making LAG3 a surrogate biomarker of active inflammation in the tumor microenvironment. Our analysis demonstrated positive correlations of a signature of IFN-γ and IFN-γ related genes with *LAG3* mRNA expression and promoter hypomethylation. Our functional data from A375 cells provided further insight in the interplay of IFN-γ, *LAG3* expression, and the methylation status. Contrary to our expectations, IFN-γ treatment alone had no stimulating effect on *LAG3* mRNA expression but, on the opposite, even showed a significant decrease of *LAG3* levels in the cell line under investigation. In the state of hypomethylation, however, IFN-γ stimulation gained the capacity to induce pronounced and significant levels of *LAG3* mRNA. IFN-γ signature and tumor-infiltrating lymphocytes have been demonstrated to be prognostic biomarkers in many types of cancer [77]. With regard to the demonstrated function of LAG3 as a surrogate biomarker for a cytotoxic anti-tumor response, we assumed *LAG3* to be predictive for outcome in melanoma patients. The results of our cell line experiments provides further functional insights indicating that *LAG3* promoter hypomethylation could be the crucial regulative mechanism within this connection, rendering tumors cells receptive for IFN-γ efficiency.

In line with the observed associations of *LAG3* methylation with lymphocyte infiltration and IFN-γ signature, our Kaplan-Meier survival analyses of the TCGA cohort confirmed the better prognosis of patients with high mRNA-expressing tumors and tumors showing hypomethylation of the CpG sites located in the promoter region. To further test the predictive significance of *LAG3* methylation, we analyzed tumor tissue obtained from metastatic melanoma patients treated with ICB with regard to progression under therapy. Kaplan-Meier survival analyses confirmed a better PFS of patients with tumors showing hypomethylation in two CpG sites located in the promoter region. Our results are in line with a current meta-analysis, which demonstrated an association of LAG3 expression with better outcome in diverse tumor entities [30].

Given the immunosuppressive effects of inhibitory immune checkpoints including PD-1, CTLA-4, and LAG3, and their published role in tumor escape this association seems paradoxical [30]. Immune checkpoint signaling is known to impair T cell proliferation, decrease cytokine secretion, lead to exhausted TILs, and in turn induce an immune suppressive signal, including the tracking of Tregs [6]. High levels of Tregs are associated with poorer prognosis and reduced overall survival [78]. In the early phase, however, the expression of immune checkpoint receptors might reflect the initial interplay between tumor and immune cells and the subsequent active immune response – a stage also referred to as “hot tumor” [79]. Indeed, PD-1 and LAG3 are commonly co-expressed upon immune activation and are associated with the expression of CD8 [22,24], which is an accepted prognostic biomarker [80]. PD-L1 expression has been discussed as a surrogate biomarker for the presence of an immune-active environment [81]. In accordance with our results, a current study suggested *PD-L1* promoter methylation as an independent survival prognostic factor in melanoma and uncovered associations of *PD‐L1* methylation with an “interferon signaling transcriptional phenotype” [43]. Further studies reported similar associations between the expression of PD-L1 and CTLA-4 with improved outcome in diverse malignancies [82], [83], [84]. Based on our results, we suggest the feasibility of *LAG3* methylation to reflect anti-tumor response. Beyond, *LAG3* methylation needs to be considered a valuable prognostic biomarker and in future could even have therapeutic applications in melanoma.

Our study has some limitations. A recent meta-analysis on the prognostic value of LAG3 in cancer proposed LAG3 expression to be associated with better overall survival, with a trend towards higher benefit in early stage cancer than in metastatic disease [30]. The TCGA cohort only provides data on tumor stage by time of the primary diagnosis, thus limiting correlation analyses of survival with tumor stage. To avoid the potential bias generated by tumor stage we validated the predictive significance of *LAG3* methylation in the ICB cohort, which is exclusively composed of stage IV patients. For correlation analyses of methylation levels in immune cells we used PBMCs derived from healthy donors which might differ from immune cells in cancer patients due to a lack of tumor cell antigen exposure. Beyond, leukocyte DNA methylation has been shown to differ by ethnicity, gender, aging, demographics, and environmental factors [85], limiting the possibility of building a homogenous comparative group. As our study aimed at investigating an epigenetic regulation of *LAG3* expression via DNA methylation, drawing further conclusions would be highly speculative. We therefore didn't conclude which CpG site might be ultimately used as a biomarker. One of the main results of our study is that CpGs sites sequence-contextually indicate distinct information and needs to be considered individually as a biomarker. The TCGA cohort does not include data on LAG3 protein expression on single immune cell subsets. In our study, we used RNA-Seq data of tumor infiltrating leukocytes as a biostatistical approach of tissue deconvolution instead. Our analysis did not account for isoform specific expression analysis, even though isoform specific transcription analyses on TILs might provide valuable information and should be followed up in future studies.

The use of DNA methylation in the experimental and clinical setting has some advantages. The analysis of TILs demands immunohistochemistry or RNA-Seq, with the latter being limited in FFPET. Beyond, LAG3 is not expressed constitutively. Activation-dependent expression on CD4^+^ cells was detected 24 hours after in vitro stimulation. In the course of time, metalloproteases cleave LAG3 from LAG3^+^ cells in a negative feedback loop [5,86]. Here, DNA methylation constitutes a rather time and tissue independent robust additional diagnostic tool. In view of our results, we assume *LAG3* methylation to be a crucial regulative mechanism of *LAG3* expression and to be a sensible prognostic biomarker reflecting the complex molecular interplay within the tumor microenvironment. Wu et al. recently identified the predictive significance of mutations in a DNA demethylase in cancer patients undergoing ICB treatment [87], pointing out the biologic relevance of methylation analyses for individual treatment planning. Effectively, immune checkpoint gene expression, density of tumor infiltrating lymphocytes (TILs), and mutational load have been identified as biomarkers for immune checkpoint blocking molecules [73]. However, despite its value as prognostic biomarkers, immune checkpoint mRNA expression and IFN-γ have so far not sufficiently proven their suitability as predictive biomarkers for patients treated with immunotherapy [88]. In view of the promising results from our ICB cohort showing value of *LAG3* methylation to predict PFS, we see the potential of *LAG3* methylation to serve as a predictive biomarker for response to anti-LAG3 antibodies and therefore recommend to further evaluate this hypothesis in biomarker programs of clinical trials.

The primary focus of our study was on the epigenetic regulation of *LAG3* in melanoma. In conclusion, our results suggest that *LAG3* mRNA expression is regulated via DNA methylation. We validated melanoma cell-intrinsic *LAG3* expression and a transcriptional regulation via DNA methylation in the established melanoma cell line A375. Beyond, the demonstrated correlations of *LAG3* DNA methylation with known clinicopathological and molecular features of immune response provide first evidence of *LAG3* methylation as a potential prognostic and predictive biomarker in melanoma patients. Based on our results, we suggest following up on *LAG3* DNA methylation as a biomarker in melanoma patients and to test the predictive value of *LAG3* DNA methylation in patients treated with LAG3 targeted antibodies.

## Declaration of Competing Interests

DD owns patents and patent aplications on methylation of immune checkpoint genes (including *LAG3*) as predictive and prognostic biomarkers (DE102017125780, DE102016005947, DE102015009187, WO2019086642, US2019249258, CN109715829, EP3458602, JP2019516383, WO2017198617, EP3322819, WO2017008912, CN108138242). The patents are licensed to Qiagen GmbH (Hilden, Germany) and DD receives royalties from Qiagen. DD is a consultant for AJ Innuscreen GmbH (Berlin, Germany), a 100% daughter company of Analytik Jena AG (Jena, Germany), and receives royalties from product sales (innuCONVERT kits). JS received speaker's honoraria and/or travel reimbursements from Novartis, Bristol-Myers Squibb, Merck Sharp and Dohme, Roche, and Pierre Fabre. AF has received speaker's honoraria or travel expense reimbursements from the following companies: Novartis, Bristol-Myers Squibb, Almirall, and Eli Lilly Pharma. JL is a consultant / advisory board member of Bristol-Myers Squibb, Merck, Novartis, and Roche. FH has received speakers’ honoraria or travel expense reimbursements from the following companies: Roche, BMS, and Novartis. LF reported grants from the Swiss National Science Foundation, Hookipa Pharma, Krebsliga Schweiz, and Novartis Foundation as well as an advisory role for Novartis, Sanofi and Bristol-Myers Squibb. No potential conflicts of interest were disclosed by the other authors.

## Appendix Supplementary materials

**Supplemental Table 1.** Associations and correlations of *LAG3* methylation and mRNA expression with clinicopathologic parameters, molecular features and immune cell infiltrates in *N*\elsamp #x00A0;\elsamp #x003D;\elsamp #x00A0;468 melanoma patients from The Cancer Genome Atlas. Molecular data were adopted from The Cancer Genome Atlas [49]. *P*-values refer to Kruskal-Wallis (\elsamp #x003E; two group comparisons), Wilcoxon Mann-Whitney *U* (2 group comparison) tests, and Spearman\elsamp #x0027;s rank correlation (continuous variables), respectively. Image, application 1

**Supplemental Table 2.** Results from pairwise Bonferroni post-hoc comparisons of *LAG3* methylation at 16 CpG sites in isolated leucocytes (*N*\elsamp #x00A0;\elsamp #x003D;\elsamp #x00A0;53 monocytes, *N*\elsamp #x00A0;\elsamp #x003D;\elsamp #x00A0;60 B cells, *N*\elsamp #x00A0;\elsamp #x003D;\elsamp #x00A0;24 CD8^\elsamp #x002B;^ T cells, *N*\elsamp #x00A0;\elsamp #x003D;\elsamp #x00A0;97 CD4^\elsamp #x002B;^ T cells, and *N*\elsamp #x00A0;\elsamp #x003D;\elsamp #x00A0;18 regulatory T cells) from healthy donors, melanocytes (*N*\elsamp #x00A0;\elsamp #x003D;\elsamp #x00A0;23) and melanoma cell lines (*N*\elsamp #x00A0;\elsamp #x003D;\elsamp #x00A0;16). ANOVA *P* \elsamp #x003C; 0.001 for all 16 sites. Image, application 2
